# 2-Oxo-2-(2-oxo-2*H*-chromen-3-yl)ethyl di­ethyl­dithio­carbamate

**DOI:** 10.1107/S1600536813021806

**Published:** 2013-08-21

**Authors:** T. G. Meenakshi, H. C. Devarajegowda, K. Mahesh Kumar, O. Kotresh, Venkatesh B. Devaru

**Affiliations:** a Department of Physics, Y. Y. D. Govt. First Grade College, Belur 573 115, Hassan, Karnataka, India; b Department of Physics, Yuvaraja’s College (Constituent College), University of Mysore, Mysore 570 005, Karnataka, India; c Department of Chemistry, Karnatak University’s Karnatak Science College, Dharwad, Karnataka 580 001, India; d P. G. Department of Physics, LVD College, Raichur 584 103, Karnataka, India

## Abstract

In the title compound, C_16_H_17_NO_3_S_2_, the dihedral angles between the O/C/C/S group and the 2*H*-chromene ring system and the thio­carbamate group are 14.46 (9) and 83.30 (9)°, respectively. The bond-angle sum at the N atom is 360.0°. One of the methyl C atoms lies above the thio­carbamate plane and one lies below it [deviations = 1.264 (3) and −1.147 (3) Å, respectively]. In the crystal, inversion dimers linked by pairs of C—H⋯O hydrogen bonds generate *R*_2_^2^(10) loops. Weak aromatic π–π stacking inter­actions [shortest centroid–centroid distance = 3.8138 (11) Å] are also observed.

## Related literature

For backgrond to chromenes, a related structure and the synthesis of the title compound, see: Kumar *et al.* (2012 ▶).
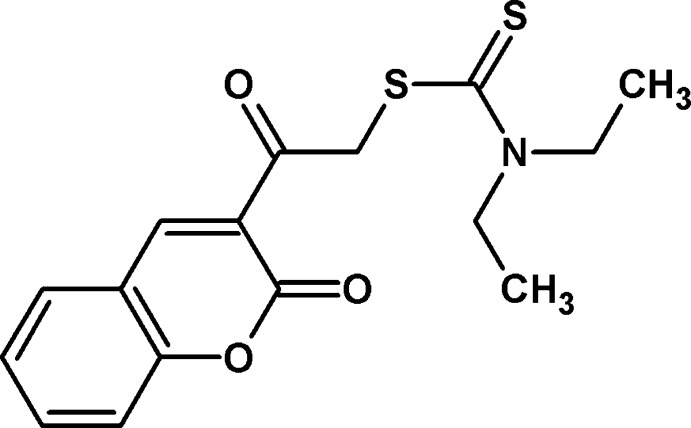


## Experimental

### 

#### Crystal data

C_16_H_17_NO_3_S_2_
*M* *_r_* = 335.43
Orthorhombic, 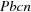

*a* = 16.3379 (5) Å
*b* = 9.6445 (3) Å
*c* = 20.5078 (6) Å
*V* = 3231.43 (17) Å^3^
*Z* = 8
Mo *K*α radiation
μ = 0.34 mm^−1^
*T* = 296 K
0.24 × 0.20 × 0.12 mm

#### Data collection

Bruker SMART CCD diffractometer
Absorption correction: ψ scan (*SADABS*; Bruker, 2001 ▶) *T*_min_ = 0.770, *T*_max_ = 1.000
12047 measured reflections
2831 independent reflections
2129 reflections with *I* > 2σ(*I*)
*R*_int_ = 0.030

#### Refinement

*R*[*F* ^2^ > 2σ(*F* ^2^)] = 0.035
*wR*(*F* ^2^) = 0.090
*S* = 1.05
2831 reflections
199 parameters
H-atom parameters constrained
Δρ_max_ = 0.21 e Å^−3^
Δρ_min_ = −0.18 e Å^−3^

### 

Data collection: *SMART* (Bruker, 2001 ▶); cell refinement: *SAINT* (Bruker, 2001 ▶); data reduction: *SAINT*; program(s) used to solve structure: *SHELXS97* (Sheldrick, 2008 ▶); program(s) used to refine structure: *SHELXL97* (Sheldrick, 2008 ▶); molecular graphics: *ORTEP-3 for Windows* (Farrugia, 2012 ▶); software used to prepare material for publication: *SHELXL97*.

## Supplementary Material

Crystal structure: contains datablock(s) I, global. DOI: 10.1107/S1600536813021806/hb7115sup1.cif

Structure factors: contains datablock(s) I. DOI: 10.1107/S1600536813021806/hb7115Isup2.hkl

Supplementary material file. DOI: 10.1107/S1600536813021806/hb7115Isup3.cml

Additional supplementary materials:  crystallographic information; 3D view; checkCIF report

## Figures and Tables

**Table 1:** Hydrogen-bond geometry (Å, °)

*D*—H⋯*A*	*D*—H	H⋯*A*	*D*⋯*A*	*D*—H⋯*A*
C9—H9⋯O5^i^	0.93	2.49	3.198 (2)	134

Symmetry code: (i) 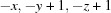.

**Fig. 1.**
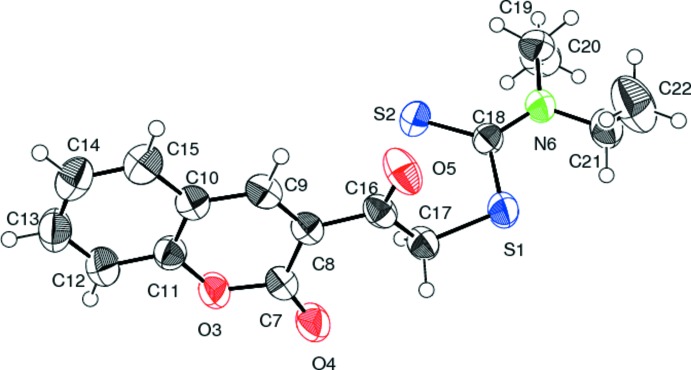
The molecular structure of the title compound. Displacement ellipsoids are drawn at the 50% probability level.

**Fig. 2.**
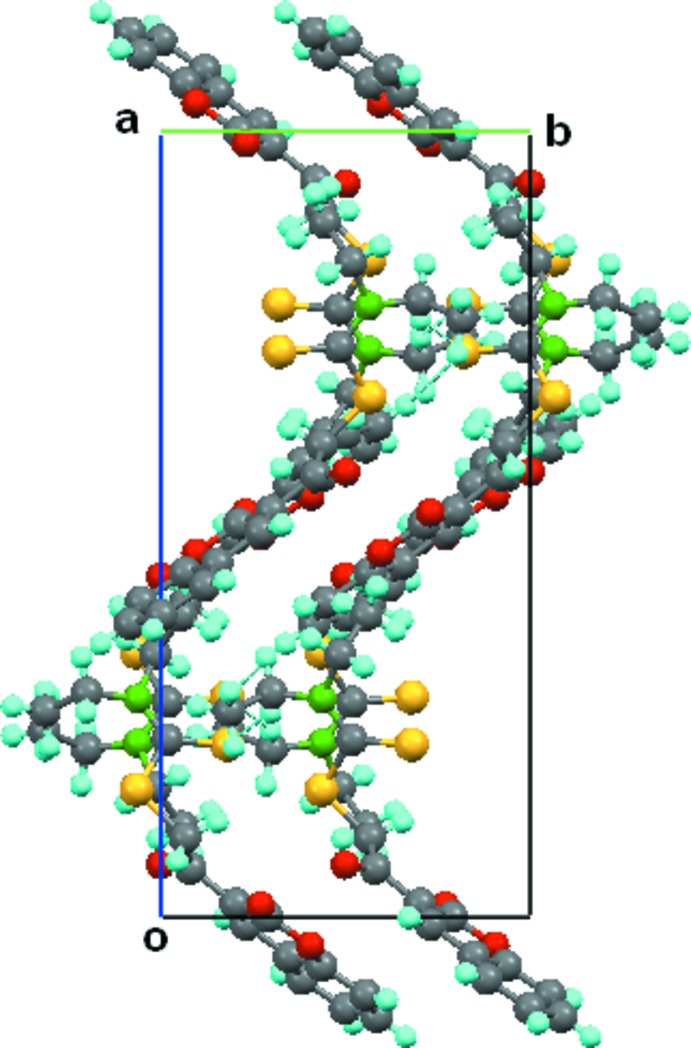
The packing of molecule shows when view along c axis.

## supplementary crystallographic information

### 1. Comment

As part of our ongoing structural studies of chromene derivatives with possible biological activity (Kumar *et al.*, 2012), we now describe the structure of the title compound, (I), (Fig. 1).

The 2*H*-chromene ring system is close to planar, with a maximum deviation of 0.031 (1) Å for atom C8. In the crystal, C9—H9···O5 hydrogen bonds (Table 1) and π-π interactions between fused benzene rings of chromene [shortest centroid–centroid distance = 3.8138 (11) Å] occur. The C—H···O hydrogen bonds generate an *R*_2_^2^(10) loop.

### 2. Experimental

This compound was prepared according to the reported method (Kumar *et al.*, 2012). Colourless blocks of the title compound were grown from a mixed solution of EtOH/CHCl_3_ (V/V = 2/1) by slow evaporation at room temperature. Yield= 90%, m.p. 380 K.

### 3. Refinement

All H atoms were positioned geometrically, with C—H = 0.93 Å for aromatic H, C—H = 0.97 Å for methylene H and C—H = 0.96 Å for methyl H,and refined using a riding model with *U*_iso_(H) = 1.5*U*_eq_(C) for methyl H and *U*_iso_(H) = 1.2*U*_eq_(C) for all other H.

### Crystal data

**Table:**

C_16_H_17_NO_3_S_2_	*D*_x_ = 1.379 Mg m^−^^3^
*M**_r_* = 335.43	Melting point: 380 K
Orthorhombic, *P**b**c**n*	Mo *K*α radiation, λ = 0.71073 Å
Hall symbol: -P 2n 2ab	Cell parameters from 2831 reflections
*a* = 16.3379 (5) Å	θ = 2.0–25.0°
*b* = 9.6445 (3) Å	µ = 0.34 mm^−^^1^
*c* = 20.5078 (6) Å	*T* = 296 K
*V* = 3231.43 (17) Å^3^	Block, colourless
*Z* = 8	0.24 × 0.20 × 0.12 mm
*F*(000) = 1408	

### Data collection

**Table:**

Bruker SMART CCD diffractometer	2831 independent reflections
Radiation source: fine-focus sealed tube	2129 reflections with *I* > 2σ(*I*)
Graphite monochromator	*R*_int_ = 0.030
ω and φ scans	θ_max_ = 25.0°, θ_min_ = 2.0°
Absorption correction: ψ scan (*SADABS*; Bruker, 2001)	*h* = −19→16
*T*_min_ = 0.770, *T*_max_ = 1.000	*k* = −11→11
12047 measured reflections	*l* = −21→23

### Refinement

**Table:**

Refinement on *F*^2^	Primary atom site location: structure-invariant direct methods
Least-squares matrix: full	Secondary atom site location: difference Fourier map
*R*[*F*^2^ > 2σ(*F*^2^)] = 0.035	Hydrogen site location: inferred from neighbouring sites
*wR*(*F*^2^) = 0.090	H-atom parameters constrained
*S* = 1.05	*w* = 1/[σ^2^(*F*_o_^2^) + (0.0414*P*)^2^ + 0.5615*P*] where *P* = (*F*_o_^2^ + 2*F*_c_^2^)/3
2831 reflections	(Δ/σ)_max_ = 0.001
199 parameters	Δρ_max_ = 0.21 e Å^−^^3^
0 restraints	Δρ_min_ = −0.18 e Å^−^^3^

### Special details

**Table:**

Experimental. IR (KBr): 634 cm^-1^ (C—S), 1266 cm^-1^ (C=S), 1069 cm^-1^ (C—O), 859 cm^-1^ (C—N),1170 cm^-1^ (C—O—C), 1696 cm^-1^ (C=O), 1725 cm^-1^ (Coumarin C=O). GCMS: m/e: 335. 1H NMR (400 MHz, CDCl_3_, \?,. p.p.m): 1.24 (m, 3H, C_12_), 1.35 (m, 3H, C_1_), 3.80 (t, 2H, C_2_),3.97(t, 2H, C_13_), 4.80(s,2*H*, C_4_), 7.27 (s, 1H, C_16_), 7.37 (m, 1H, C_10_),7.66 (s, 1H, C_11_), 8.49(s, 1H, C_9_).13 C NMR (400 MHz, CDCl3, \?,. p.p.m): 194(C_3_), 191(C_5_), 159(C_14_), 155(C_15_), 147(C_7_), 134(C_6_), 130(C_11_), 125(C_9_), 125(C_10_), 118(C_8_), 116(C_16_), 50(C_4_), 47(C_2_),46(C_13_), 12(C_1_), 11(C_12_).Elemental analysis for C_16_H_17_NO_3_S_2_: C, 57.22; H, 5.06; N, 4.11.
Geometry. All s.u.'s (except the s.u. in the dihedral angle between two l.s. planes) are estimated using the full covariance matrix. The cell s.u.'s are taken into account individually in the estimation of s.u.'s in distances, angles and torsion angles; correlations between s.u.'s in cell parameters are only used when they are defined by crystal symmetry. An approximate (isotropic) treatment of cell s.u.'s is used for estimating s.u.'s involving l.s. planes.
Refinement. Refinement of *F*^2^ against ALL reflections. The weighted *R*-factor *wR* and goodness of fit *S* are based on *F*^2^, conventional *R*-factors *R* are based on *F*, with *F* set to zero for negative *F*^2^. The threshold expression of *F*^2^ > 2σ(*F*^2^) is used only for calculating *R*-factors(gt) *etc*. and is not relevant to the choice of reflections for refinement. *R*-factors based on *F*^2^ are statistically about twice as large as those based on *F*, and *R*- factors based on ALL data will be even larger.

### Fractional atomic coordinates and isotropic or equivalent isotropic displacement parameters (Å^2^)

**Table:**

	*x*	*y*	*z*	*U*_iso_*/*U*_eq_	
S1	0.20407 (3)	0.56622 (6)	0.33761 (2)	0.05998 (19)	
S2	0.11615 (3)	0.32066 (6)	0.28013 (3)	0.05692 (18)	
O3	0.17542 (8)	0.09457 (14)	0.53601 (7)	0.0570 (4)	
O4	0.26174 (8)	0.23027 (17)	0.48512 (7)	0.0714 (5)	
O5	0.07374 (9)	0.50120 (16)	0.43332 (7)	0.0685 (4)	
N6	0.13841 (10)	0.56284 (17)	0.22176 (7)	0.0501 (4)	
C7	0.19123 (12)	0.2115 (2)	0.49934 (9)	0.0490 (5)	
C8	0.12118 (10)	0.2984 (2)	0.48279 (8)	0.0417 (4)	
C9	0.04753 (11)	0.2690 (2)	0.50922 (9)	0.0468 (5)	
H9	0.0038	0.3278	0.5004	0.056*	
C10	0.03396 (11)	0.1524 (2)	0.54993 (9)	0.0449 (5)	
C11	0.09952 (12)	0.0653 (2)	0.56141 (9)	0.0481 (5)	
C12	0.09278 (14)	−0.0535 (2)	0.59863 (11)	0.0628 (6)	
H12	0.1376	−0.1114	0.6050	0.075*	
C13	0.01817 (15)	−0.0843 (2)	0.62606 (11)	0.0658 (6)	
H13	0.0126	−0.1631	0.6518	0.079*	
C14	−0.04852 (14)	0.0007 (3)	0.61575 (11)	0.0631 (6)	
H14	−0.0987	−0.0217	0.6344	0.076*	
C15	−0.04160 (12)	0.1179 (2)	0.57822 (10)	0.0577 (5)	
H15	−0.0869	0.1744	0.5715	0.069*	
C16	0.12926 (12)	0.4194 (2)	0.43773 (9)	0.0468 (5)	
C17	0.20748 (12)	0.4352 (2)	0.39882 (9)	0.0517 (5)	
H17A	0.2200	0.3473	0.3782	0.062*	
H17B	0.2518	0.4563	0.4287	0.062*	
C18	0.14928 (10)	0.4828 (2)	0.27391 (9)	0.0445 (5)	
C19	0.08964 (13)	0.5153 (3)	0.16571 (9)	0.0585 (6)	
H19A	0.0624	0.5941	0.1459	0.070*	
H19B	0.0478	0.4515	0.1808	0.070*	
C20	0.14183 (16)	0.4445 (3)	0.11560 (11)	0.0759 (7)	
H20A	0.1082	0.4155	0.0797	0.114*	
H20B	0.1677	0.3650	0.1348	0.114*	
H20C	0.1829	0.5077	0.1003	0.114*	
C21	0.17643 (16)	0.7006 (2)	0.21421 (11)	0.0694 (7)	
H21A	0.1894	0.7154	0.1686	0.083*	
H21B	0.2273	0.7027	0.2385	0.083*	
C22	0.1227 (2)	0.8147 (3)	0.23727 (15)	0.1102 (11)	
H22A	0.1500	0.9019	0.2312	0.165*	
H22B	0.1108	0.8019	0.2827	0.165*	
H22C	0.0726	0.8141	0.2128	0.165*	

### Atomic displacement parameters (Å^2^)

**Table:**

	*U*^11^	*U*^22^	*U*^33^	*U*^12^	*U*^13^	*U*^23^
S1	0.0785 (4)	0.0517 (4)	0.0498 (3)	−0.0153 (3)	−0.0134 (3)	0.0112 (3)
S2	0.0549 (3)	0.0488 (3)	0.0670 (4)	−0.0095 (2)	0.0006 (2)	0.0070 (3)
O3	0.0494 (8)	0.0523 (9)	0.0693 (9)	0.0053 (6)	−0.0001 (7)	0.0199 (8)
O4	0.0480 (8)	0.0799 (12)	0.0864 (11)	0.0065 (8)	0.0037 (7)	0.0350 (9)
O5	0.0805 (10)	0.0611 (10)	0.0639 (9)	0.0304 (9)	0.0120 (8)	0.0156 (8)
N6	0.0563 (9)	0.0491 (11)	0.0450 (9)	−0.0060 (8)	−0.0037 (7)	0.0071 (8)
C7	0.0536 (12)	0.0485 (13)	0.0448 (10)	0.0028 (10)	−0.0023 (9)	0.0057 (10)
C8	0.0506 (11)	0.0390 (11)	0.0356 (9)	0.0048 (9)	−0.0019 (8)	−0.0038 (8)
C9	0.0534 (11)	0.0435 (12)	0.0434 (10)	0.0095 (9)	−0.0031 (9)	−0.0056 (10)
C10	0.0523 (11)	0.0425 (12)	0.0400 (10)	0.0011 (9)	0.0001 (8)	−0.0056 (9)
C11	0.0524 (11)	0.0464 (13)	0.0455 (11)	−0.0041 (10)	−0.0007 (9)	0.0004 (10)
C12	0.0667 (14)	0.0510 (14)	0.0707 (14)	−0.0007 (11)	−0.0040 (11)	0.0134 (12)
C13	0.0846 (17)	0.0540 (15)	0.0588 (13)	−0.0160 (13)	0.0022 (12)	0.0081 (12)
C14	0.0659 (14)	0.0632 (16)	0.0602 (13)	−0.0158 (13)	0.0136 (11)	−0.0075 (12)
C15	0.0561 (12)	0.0583 (14)	0.0586 (12)	−0.0002 (10)	0.0072 (10)	−0.0059 (12)
C16	0.0591 (12)	0.0428 (12)	0.0384 (10)	0.0064 (10)	−0.0055 (9)	−0.0019 (9)
C17	0.0594 (12)	0.0510 (13)	0.0445 (10)	−0.0019 (10)	−0.0054 (9)	0.0073 (10)
C18	0.0390 (9)	0.0486 (12)	0.0460 (11)	0.0000 (9)	0.0043 (8)	0.0051 (10)
C19	0.0561 (11)	0.0673 (15)	0.0522 (12)	−0.0056 (11)	−0.0109 (10)	0.0059 (12)
C20	0.0916 (17)	0.0818 (19)	0.0544 (13)	0.0017 (14)	−0.0066 (12)	−0.0065 (13)
C21	0.0908 (16)	0.0625 (16)	0.0550 (13)	−0.0156 (13)	−0.0106 (12)	0.0138 (12)
C22	0.177 (3)	0.068 (2)	0.085 (2)	0.024 (2)	−0.017 (2)	0.0027 (17)

### Geometric parameters (Å, º)

**Table:**

S1—C18	1.7762 (19)	C13—C14	1.380 (3)
S1—C17	1.7818 (19)	C13—H13	0.9300
S2—C18	1.660 (2)	C14—C15	1.372 (3)
O3—C11	1.374 (2)	C14—H14	0.9300
O3—C7	1.380 (2)	C15—H15	0.9300
O4—C7	1.202 (2)	C16—C17	1.514 (3)
O5—C16	1.206 (2)	C17—H17A	0.9700
N6—C18	1.331 (2)	C17—H17B	0.9700
N6—C19	1.472 (2)	C19—C20	1.500 (3)
N6—C21	1.474 (3)	C19—H19A	0.9700
C7—C8	1.459 (3)	C19—H19B	0.9700
C8—C9	1.350 (2)	C20—H20A	0.9600
C8—C16	1.494 (3)	C20—H20B	0.9600
C9—C10	1.418 (3)	C20—H20C	0.9600
C9—H9	0.9300	C21—C22	1.485 (4)
C10—C11	1.381 (3)	C21—H21A	0.9700
C10—C15	1.404 (3)	C21—H21B	0.9700
C11—C12	1.382 (3)	C22—H22A	0.9600
C12—C13	1.375 (3)	C22—H22B	0.9600
C12—H12	0.9300	C22—H22C	0.9600
			
C18—S1—C17	102.29 (9)	C8—C16—C17	118.65 (16)
C11—O3—C7	122.91 (15)	C16—C17—S1	114.62 (14)
C18—N6—C19	121.27 (17)	C16—C17—H17A	108.6
C18—N6—C21	123.41 (16)	S1—C17—H17A	108.6
C19—N6—C21	115.27 (16)	C16—C17—H17B	108.6
O4—C7—O3	115.77 (17)	S1—C17—H17B	108.6
O4—C7—C8	127.52 (19)	H17A—C17—H17B	107.6
O3—C7—C8	116.71 (16)	N6—C18—S2	124.38 (14)
C9—C8—C7	119.04 (18)	N6—C18—S1	113.30 (14)
C9—C8—C16	119.41 (17)	S2—C18—S1	122.31 (11)
C7—C8—C16	121.55 (16)	N6—C19—C20	111.66 (17)
C8—C9—C10	122.84 (17)	N6—C19—H19A	109.3
C8—C9—H9	118.6	C20—C19—H19A	109.3
C10—C9—H9	118.6	N6—C19—H19B	109.3
C11—C10—C15	117.86 (19)	C20—C19—H19B	109.3
C11—C10—C9	117.47 (17)	H19A—C19—H19B	107.9
C15—C10—C9	124.65 (18)	C19—C20—H20A	109.5
O3—C11—C10	120.69 (17)	C19—C20—H20B	109.5
O3—C11—C12	116.85 (18)	H20A—C20—H20B	109.5
C10—C11—C12	122.46 (19)	C19—C20—H20C	109.5
C13—C12—C11	118.4 (2)	H20A—C20—H20C	109.5
C13—C12—H12	120.8	H20B—C20—H20C	109.5
C11—C12—H12	120.8	N6—C21—C22	112.7 (2)
C12—C13—C14	120.6 (2)	N6—C21—H21A	109.1
C12—C13—H13	119.7	C22—C21—H21A	109.1
C14—C13—H13	119.7	N6—C21—H21B	109.1
C15—C14—C13	120.7 (2)	C22—C21—H21B	109.1
C15—C14—H14	119.7	H21A—C21—H21B	107.8
C13—C14—H14	119.7	C21—C22—H22A	109.5
C14—C15—C10	120.0 (2)	C21—C22—H22B	109.5
C14—C15—H15	120.0	H22A—C22—H22B	109.5
C10—C15—H15	120.0	C21—C22—H22C	109.5
O5—C16—C8	119.41 (18)	H22A—C22—H22C	109.5
O5—C16—C17	121.94 (18)	H22B—C22—H22C	109.5
			
C11—O3—C7—O4	173.66 (18)	C13—C14—C15—C10	−0.1 (3)
C11—O3—C7—C8	−5.8 (3)	C11—C10—C15—C14	−0.1 (3)
O4—C7—C8—C9	−172.6 (2)	C9—C10—C15—C14	178.54 (19)
O3—C7—C8—C9	6.8 (3)	C9—C8—C16—O5	11.3 (3)
O4—C7—C8—C16	6.9 (3)	C7—C8—C16—O5	−168.29 (18)
O3—C7—C8—C16	−173.64 (15)	C9—C8—C16—C17	−168.69 (17)
C7—C8—C9—C10	−3.5 (3)	C7—C8—C16—C17	11.7 (3)
C16—C8—C9—C10	176.94 (17)	O5—C16—C17—S1	−9.5 (3)
C8—C9—C10—C11	−1.1 (3)	C8—C16—C17—S1	170.48 (13)
C8—C9—C10—C15	−179.82 (18)	C18—S1—C17—C16	−78.83 (16)
C7—O3—C11—C10	1.4 (3)	C19—N6—C18—S2	4.5 (3)
C7—O3—C11—C12	−178.37 (18)	C21—N6—C18—S2	−172.67 (17)
C15—C10—C11—O3	−178.91 (17)	C19—N6—C18—S1	−175.84 (14)
C9—C10—C11—O3	2.3 (3)	C21—N6—C18—S1	7.0 (2)
C15—C10—C11—C12	0.8 (3)	C17—S1—C18—N6	179.60 (14)
C9—C10—C11—C12	−177.98 (19)	C17—S1—C18—S2	−0.70 (14)
O3—C11—C12—C13	178.51 (18)	C18—N6—C19—C20	−91.4 (2)
C10—C11—C12—C13	−1.2 (3)	C21—N6—C19—C20	86.0 (2)
C11—C12—C13—C14	1.0 (3)	C18—N6—C21—C22	−93.0 (2)
C12—C13—C14—C15	−0.3 (3)	C19—N6—C21—C22	89.7 (2)

### Hydrogen-bond geometry (Å, º)

**Table:**

*D*—H···*A*	*D*—H	H···*A*	*D*···*A*	*D*—H···*A*
C9—H9···O5^i^	0.93	2.49	3.198 (2)	134

Symmetry code: (i) −*x*, −*y*+1, −*z*+1.
